# The mTORC1–G9a–H3K9me2 axis negatively regulates autophagy in fatty acid–induced hepatocellular lipotoxicity

**DOI:** 10.1016/j.jbc.2023.102937

**Published:** 2023-01-21

**Authors:** Arjamand Mushtaq, Nissar Ul Ashraf, Mohammad Altaf

**Affiliations:** Chromatin and Epigenetics Lab, Centre for Interdisciplinary Research and Innovations, University of Kashmir, Srinagar, Jammu and Kashmir, India

**Keywords:** autophagy, histone methyltransferase G9a, histone methylation, lipotoxicity, mTORC1, nonalcoholic fatty liver disease, AMPK, AMP-activated serine/threonine protein kinase, ATG, autophagy-related gene, BSA, bovine serum albumin, ChIP, chromatin immunoprecipitation, mTORC1, mammalian target of rapamycin complex 1, NAFLD, nonalcoholic fatty liver disease, qRT–PCR, quantitative RT–PCR

## Abstract

Defective autophagy and lipotoxicity are the hallmarks of nonalcoholic fatty liver disease. However, the precise molecular mechanism for the defective autophagy in lipotoxic conditions is not fully known. In the current study, we elucidated that activation of the mammalian target of rapamycin complex 1 (mTORC1)–G9a–H3K9me2 axis in fatty acid–induced lipotoxicity blocks autophagy by repressing key autophagy genes. The fatty acid–treated cells show mTORC1 activation, increased histone methyltransferase G9a levels, and suppressed autophagy as indicated by increased accumulation of the key autophagic cargo SQSTM1/p62 and decreased levels of autophagy-related proteins LC3II, Beclin1, and Atg7. Our chromatin immunoprecipitation analysis showed that decrease in autophagy was associated with increased levels of the G9a-mediated repressive H3K9me2 mark and decreased RNA polymerase II occupancy at the promoter regions of Beclin1 and Atg7 in fatty acid–treated cells. Inhibition of mTORC1 in fatty acid–treated cells decreased G9a-mediated H3K9me2 occupancy and increased polymerase II occupancy at Beclin1 and Atg7 promoters. Furthermore, mTORC1 inhibition increased the expression of Beclin1 and Atg7 in fatty acid–treated cells and decreased the accumulation of SQSTM1/p62. Interestingly, the pharmacological inhibition of G9a alone in fatty acid–treated cells decreased the H3K9me2 mark at Atg7 and Beclin1 promoters and restored the expression of Atg7 and Beclin1. Taken together, our findings have identified the mTORC1–G9a–H3K9me2 axis as a negative regulator of the autophagy pathway in hepatocellular lipotoxicity and suggest that the G9a-mediated epigenetic repression is mechanistically a key step during the repression of autophagy in lipotoxic conditions.

Macroautophagy (hereafter referred to as autophagy) is an evolutionarily conserved lysosomal degradative pathway important for survival, differentiation, development, and maintaining cellular homeostasis (1, 2, 3). In principle, autophagy plays an adaptive role to protect organisms against diverse pathologies, such as infection, cancer, neurodegeneration, aging, and metabolic diseases. Autophagy is active at basal level in most of the cells in the body, and this probably reflects its importance in the turnover of long-lived proteins and removal of damaged structures (2, 4, 5, 6). However, autophagy is upregulated by various stress situations including nutrient deficiency, growth factor withdrawal, high bioenergetic demands, oxidative stress, infection, or protein aggregate accumulation (3). Autophagy has been shown to play a context-dependent prosurvival or prodeath role by regulating or being regulated by different signaling pathways involving p53, Bif-1 (Bax-interacting factor-1), Beclin1 (BECN1), UV-RAG (ultraviolet irradiation resistance–associated gene), mTOR (mammalian target of rapamycin), protein kinase B (Akt), B-cell lymphoma 2 (Bcl-2), Ras, and class I PI3K in cancer (7, 8). Autophagic machinery is under the control of diverse signaling pathways, depending upon the nature and origin of the stimulus (9, 10, 11). Among the several components involved in the tight regulation of autophagy, mTOR complex 1 (C1) has been shown to be a key player in regulating and coordinating the respective catabolic and anabolic process in response to environmental and/or physiological stress. mTORC1 inhibition increases autophagy, whereas stimulation of mTORC1 reduces the process (6, 7). Various studies suggest that, in mammals, mTORC1 controls autophagy by regulating its various downstream effectors and various autophagy-related genes (ATGs; many of which are evolutionary conserved) that are required for the initiation and execution of autophagy (12, 13, 14). The cellular and molecular cascade how mTORC1 regulates and executes autophagy has been the subject of recent comprehensive reviews and is now emerging as a central biological pathway that functions to promote health and longevity (1, 2, 6).

Nonalcoholic fatty liver disease (NAFLD), also called as metabolic-associated fatty liver disease, is a common disorder and refers to a group of conditions ranging from simple fatty liver to steatohepatitis (fatty liver plus inflammation), fibrosis, cirrhosis, and hepatocellular carcinoma (15, 16, 17). NAFLD is a part of metabolic syndrome that results in an environment favorable for cell survival, growth, and proliferation. The presence of fibrosis in patients with NAFLD is associated with greater liver-related morbidities and mortalities (17, 18). Defective autophagy and lipotoxicity are among the important hallmarks of NAFLD. Despite a comprehensive understanding of the cellular changes that occur during the lipotoxicity in NAFLD, the molecular mechanism(s) driving these changes remain poorly understood. Recent reports suggest a connection between cellular metabolism and epigenetic mechanisms in a variety of diseases including NAFLD (19, 20). In addition, various studies have shown that obesity and lipotoxic states such as NAFLD could be the result of autophagy suppression (21, 22). The precise mechanism(s) for this autophagic suppression in lipotoxic conditions is not known. While there are many possible mechanisms, the activation of growth promoting mTORC1 in the lipotoxic states and its regulation of epigenetic processes remains an attractive hypothesis. mTORC1 has been shown to have an impact on several chromatin modifiers that play role in autophagy and cellular metabolism (23, 24). However, the specific connection/crosstalk between mTOR signaling, epigenetics, and autophagy in the lipotoxic conditions such as NAFLD has not been elucidated.

In the present study, we report that mTORC1–G9a–H3K9me2 axis negatively regulates autophagy in lipotoxic conditions. Furthermore, we identified histone methyltransferase G9a as a key epigenetic regulator of autophagy and suggested that G9a could act as a potential therapeutic target in NAFLD.

## Results

### Palmitate downregulates Atg7 and Beclin1 gene expression and blocks autophagy in hepatocellular model of lipotoxicity

**Figure 1:**
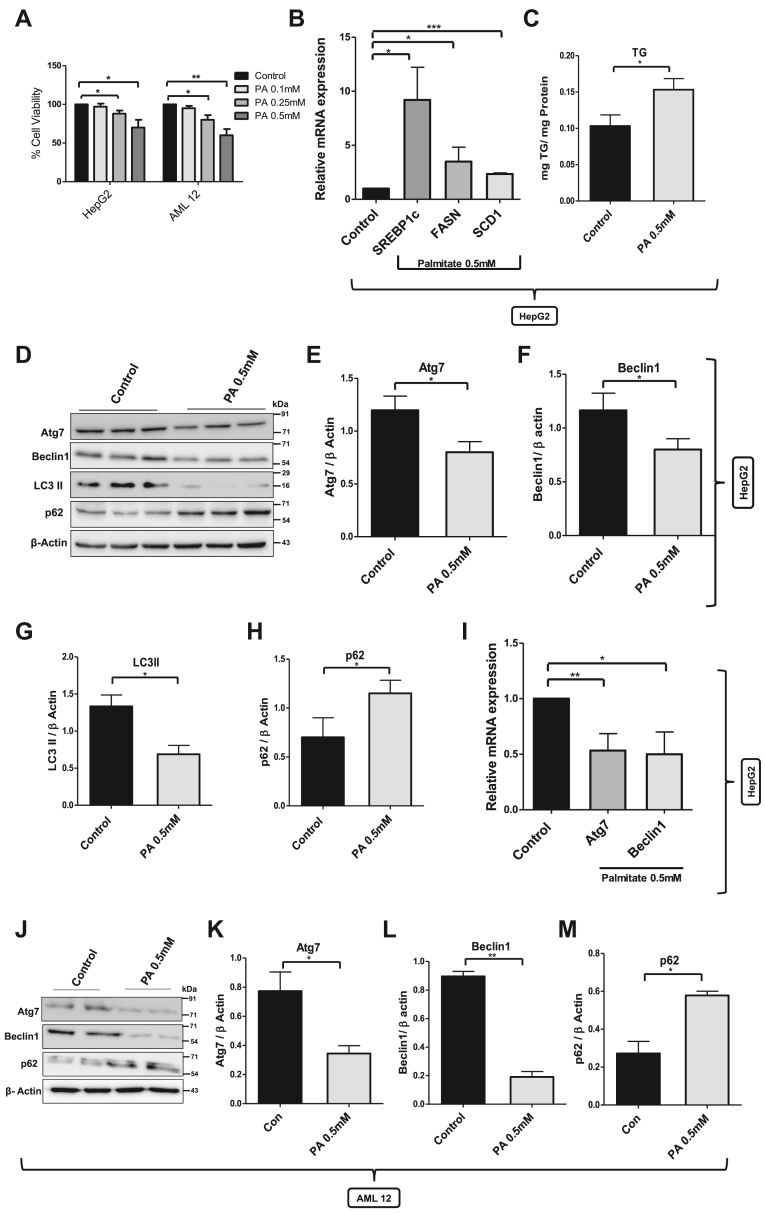
**Cytotoxicity, increased lipid accumulation, and defective autophagy in cellular model of lipotoxicity.***A,* HepG2 and AML 12 cells were treated with 0.1, 0.25, and 0.5 mM of palmitate for 24 h. Palmitate significantly induced concentration-dependent cytotoxicity in both the cell types. *B,* mRNA level of different lipogenic genes in HepG2 cells treated with 0.5 mM palmitate for 24 h (∗ indicates *p* < 0.05: control *versus* palmitate—SREBP1c and FASN, ∗∗∗∗ indicates *p* < 0.0001: control *versus* palmitate—SCD1). *C,* triglyceride accumulation in palmitate-treated HepG2 cells as measured by using LPL buffer assay (∗ indicates *p* < 0.05: control *versus* palmitate). *D,* protein level of different autophagy genes in HepG2 cells treated with 0.5 mM palmitate for 24 h. *E*–*H,* bar graphs represent the densitometric ratio of LC3II, p62, Atg7, and Beclin1 with their corresponding β-actin (∗ indicates *p* < 0.05: control *versus* palmitate). *I,* mRNA level of Atg7 and Beclin1 autophagy genes in HepG2 cells treated with 0.5 mM palmitate for 24 h (∗ indicates *p* < 0.05: control *versus* palmitate—Beclin1 and ∗∗*p* < 0.01: control *versus* palmitate—Atg7). Quantification data shown are mean ± SD. *J,* protein level of different autophagy-related genes in AML12 cells treated with 0.5 mM palmitate for 12 h. *K*–*M*, bar graphs represent the densitometric ratio of Atg7, Beclin1, and p62 with their corresponding β-actin in AML 12 cells. Error bars (wherever applicable in the figure) represent standard deviations from three independent experiments. FASN, fatty acid synthase; SREBP1c, sterol regulatory element–binding protein 1c.

Previous studies have reported that long-term exposure to free fatty acids increases lipogenesis and induces lipotoxicity in various cellular models of lipotoxicity (25). We tested whether a similar phenomenon could be recapitulated in our hepatocellular models of lipotoxicity and hence validation of our models of lipotoxicity. To test this, we treated human hepatoma cells, HepG2, with different concentrations of palmitate bound to fatty acid–free bovine serum albumin (BSA) for 24 h. The lipotoxicity was found to be concentration dependent with 10 to 20% cytotoxicity at 0.25 mM palmitate and 30 to 35% cytotoxicity at 0.5 mM (Fig. 1*A*). We also evaluated the cytotoxicity induced by free fatty acids in immortalized mouse hepatocyte AML 12 cells. AML 12 cells showed relatively more sensitivity as compared with HepG2 cells with 18 to 25% cytotoxicity at 0.25 mM palmitate and 35 to 40% at 0.5 mM (Fig. 1*A*). In order to understand the mechanism of lipotoxicity, we decided to proceed with 0.5 mM palmitate only. Next, we treated the HepG2 cells with palmitate and measured the mRNA levels of three important lipogenic genes: sterol regulatory element–binding protein 1c, fatty acid synthase, and stearoyl CoA desaturase 1. All the genes show increased expression in palmitate-treated cells as compared with untreated control cells (Fig. 1*B*). We also measured the triglyceride levels, and as expected, triglyceride levels were also increased in palmitate-treated cells (Fig. 1*C*). Increased lipogenesis and defective autophagy are the hallmarks of lipotoxicity in NAFLD. To look at the status of autophagy in palmitate-treated cells, we observed decreased LC3II/β-actin ratio (Fig. 1, *D* and *G*) and increased levels of SQSTM1/p62 (Fig. 1, *D* and *H*), a protein known to be degraded exclusively by autophagy. We also measured the protein and mRNA levels of autophagy-related genes (ARGs) in palmitate-treated cells. The mRNA as well as the protein levels of Beclin1, mammalian ortholog of Atg6 which play a central role in autophagy, and Atg7, an enzyme for the ubiquitin-like autophagy proteins, were significantly decreased in palmitate-treated cells (Fig. 1, *D*, *E*, *F* and *I*). The immortalized mouse hepatocyte AML 12 cells were also treated with 0.5 mM palmitate for 24 h. Protein levels of Atg7 and Beclin1 were significantly decreased, while as the autophagic cargo SQSTM1/p62 was increased in palmitate-treated cells as compared with untreated control cells (Fig. 1, *J*–*M*). Taken together, increased lipogenesis and lipotoxicity validated our hepatocellular model of lipotoxicity, while as the decreased levels of LC3II, Beclin1, and Atg7, and the accumulation of SQSTM1/p62 in palmitate-treated cells suggests the impairment of autophagic machinery in lipotoxic conditions.

### Activation of mTORC1–P70S6 kinase axis is associated with increased G9a-mediated repressive H3K9me2 at Atg7 and Beclin1 promoters

**Figure 2:**
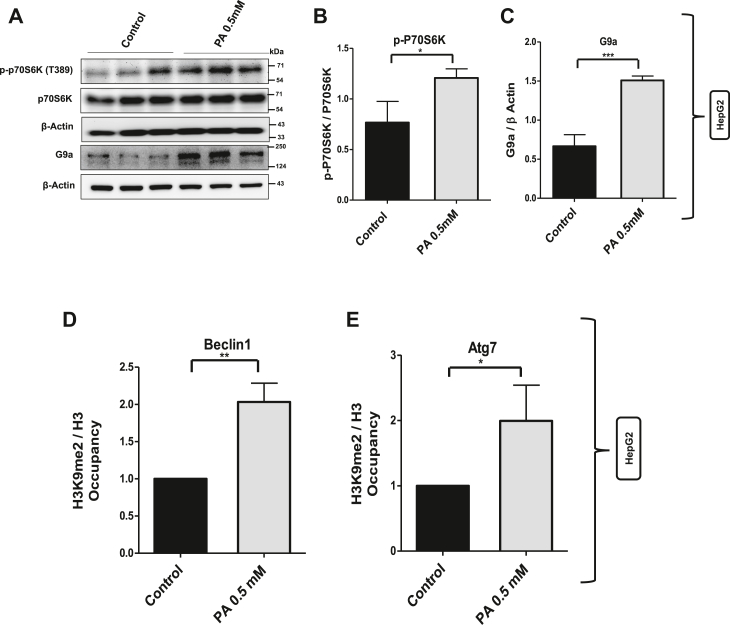
**Activation of mTORC1–P70S6 kinase axis increased G9a and increased G9a-mediated repressive H3K9me2 at Atg7 and Beclin1 promoters.***A,* immunoblotting of p-P70S6K and histone methyltransferase G9a in HepG2 cells with and without 0.5 mM palmitate for 24 h. *B* and *C,* bar graphs represent the densitometric ratio p-P70S6K with total P70S6K and G9a with its corresponding β-actin (∗ indicates *p* < 0.05). *D* and *E,* chromatin immunoprecipitation (ChIP) using H3K9me2 antibody and normalized to its corresponding total H3 followed by real-time PCR in different treatment groups as indicated in the figure for Beclin1 and Atg7 (∗ indicates *p* < 0.05). Quantification data shown are mean ± SD. Error bars (wherever applicable in the figure) represent standard deviations from three independent experiments.

**Figure 6:**
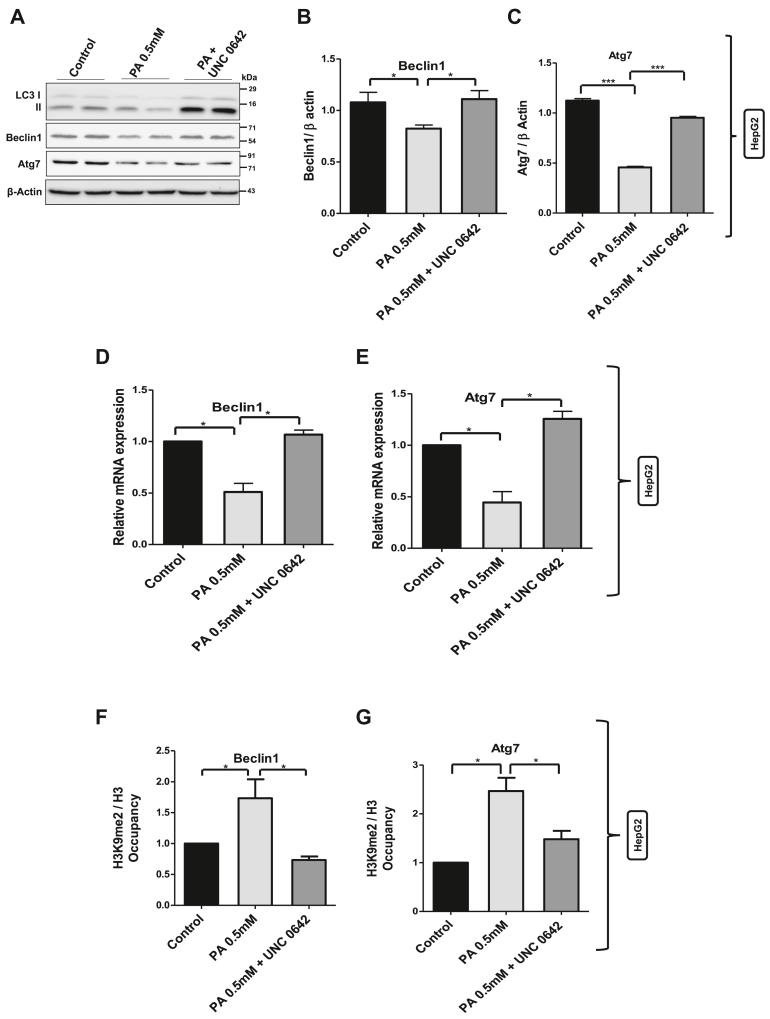

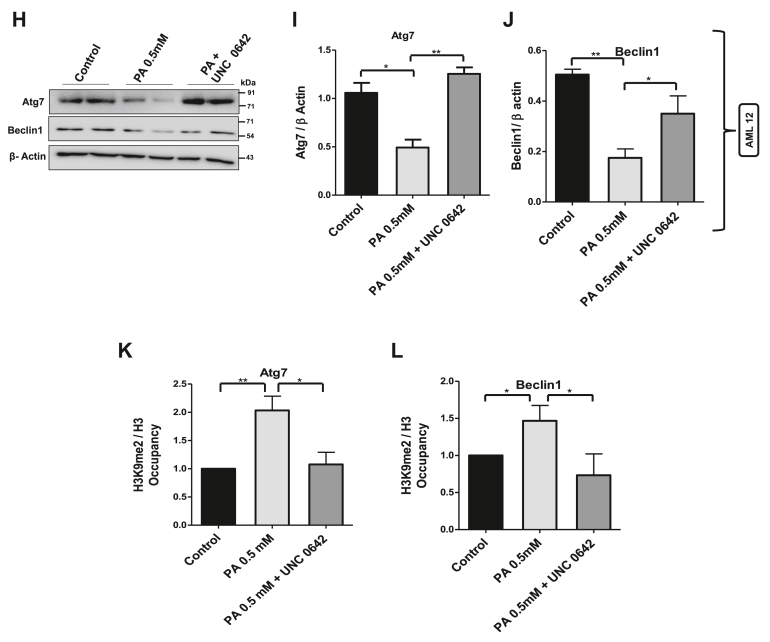
**Inhibition of G9a alone reduced the H3K9me2 occupancy and restored the expression of Atg7 and Beclin1 in lipotoxic cells.***A,* HepG2 cells treated with palmitate were pretreated with histone methyltransferase G9a inhibitor, UNC-0642 (10 μM) followed by immunoblotting using antibodies against LC3, Beclin1, and Atg7. Beta-actin was used as a loading control. *B* and *C,* bar graphs represent the densitometric ratio Beclin1 and Atg7 with their corresponding β-actin (∗ indicates *p* < 0.05, ∗∗ indicates *p* < 0.01). *D* and *E*, quantitative real-time PCR (qRT–PCR) analysis of Atg7 and Beclin1 transcript levels in different treatment groups as indicated in the figure. *F* and *G,* chromatin immunoprecipitation (ChIP) using H3K9me2 antibody and normalized to its corresponding total H3 followed by real-time PCR in different treatment groups as indicated in the figure for Atg7 and Beclin1 (∗ indicates *p* < 0.05). *H,* AML 12 cells were pretreated with G9a inhibitor UNC-0642 (10 μM) for 12 h, followed by palmitate treatment for further 12 h. Cells were lysed and subjected to immunoblotting against Atg7 and Beclin1. *I* and *J,* bar graphs representing the densitometric analysis of Atg7 and Beclin1 against their corresponding loading controls (∗ indicates *p* < 0.05, ∗∗ indicates *p* < 0.01). *K* and *L,* ChIP using H3K9me2 antibody followed by real-time PCR in different treatment groups in AML 12 cells as indicated in the figure for Atg7 and Beclin1 gene promoters (∗ indicates *p* < 0.05). Error bars (wherever applicable in the figure) represent standard deviations from three independent experiments.

mTORC1 is considered as the major gateway to autophagy. Moreover, SQSTM1/p62–mTORC1–autophagy connection has been well established, and it has been consistently reported that SQSTM1/p62 accumulates in the cells with hyperactive mTORC1 (26, 27). Furthermore, p62 has been shown to colocalize with Rags at the lysosomal compartment and is required for the interaction of mTORC1 with Rag GTPases *in vivo* and for translocation of the mTORC1 to the lysosome, an important step required for mTOR activation (26). Therefore, we studied the mTORC1 activity and measured the phosphorylation of one of the substrates of mTORC1: P70S6 kinase. Western blot analysis showed significant increase in the phosphorylation of P70S6 kinase at threonine 389 position (pP70S6K-T389) in palmitate-treated cells as compared with untreated control cells (Fig. 2, *A* and *B*). Both mTORC1 activation and histone methylation have been shown to put brakes on autophagy; therefore, we tested whether there is any association between mTORC1 activation and expression of histone methyltransferase G9a, an enzyme that methylates histone H3 at lysine 9, a mark associated with gene repression. Interestingly, the activation of mTORC1–P70S6 kinase axis in palmitate-treated HepG2 cells was associated with significant increase in the expression of histone methyltransferase G9a (Fig. 2, *A* and *C*). As mentioned previously, both mRNA and protein levels of Beclin1 and Atg7 were decreased in palmitate-treated cells (Fig. 1); therefore, we checked if the levels of repressive mark H3K9me2 at the promoter regions of Beclin1 and Atg7 genes change upon palmitate treatment. Chromatin immunoprecipitation (ChIP) analysis showed increased levels of G9a-mediated repressive H3K9me2 mark at the promoter regions of Beclin1 and Atg7 in palmitate-treated cells as compared with untreated control cells (Fig. 2, *D* and *E*). AML 12 cells also showed increase in H3K9me2 occupancy at the promoter regions of Atg7 and Beclin1 in palmitate-treated as compared with untreated control cells (Fig. 6, *K* and *L*). Collectively, these results suggest a possible connection between mTORC1 activation and G9a-mediated repressive H3K9me2 mark in lipotoxicity.

### Palmitate activates mTOR/p70S6K through AMP-activated serine/threonine protein kinase inhibition

**Figure 3:**
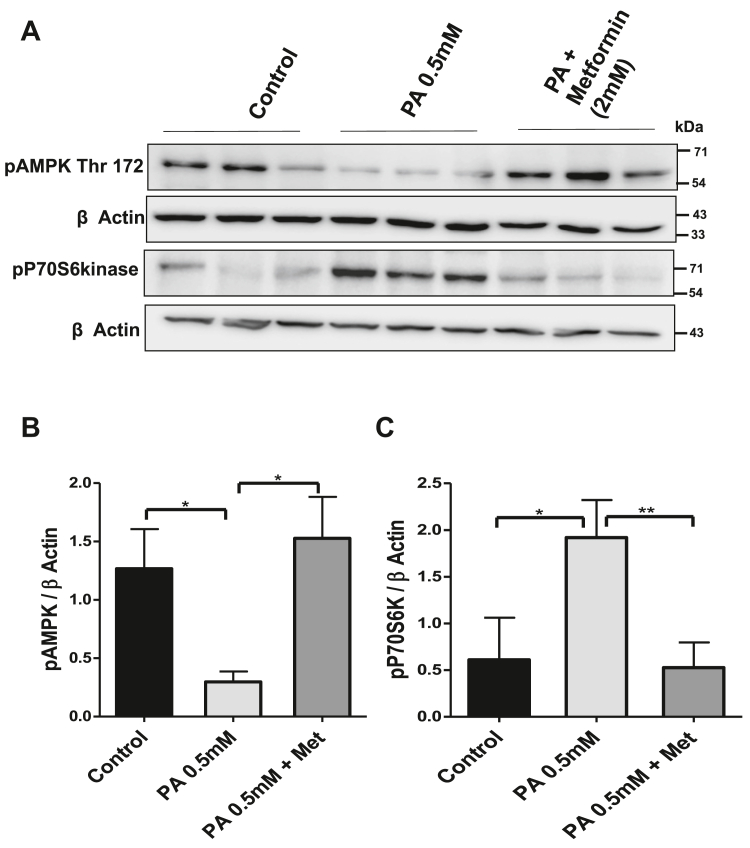
**Palmitate activates mTOR/p70S6K through AMPK inhibition.***A,* HepG2 cells treated with palmitate were pretreated (2 h) with metformin (2 mM) followed by Western blotting against pAMPK-Thr172 and mTORC1 substrate p-P70S6K in different treatment groups as indicated in the figure. *B* and *C,* bar graphs represent the densitometric ratio p-AMPK-Thr172 and p-P70S6K with their corresponding β-actin in different treatment groups (∗ indicates *p* < 0.05, ∗∗ indicates *p* < 0.01). Error bars (wherever applicable in the figure) represent standard deviations from three independent experiments. AMPK, AMP-activated serine/threonine protein kinase; mTOR, mammalian target of rapamycin.

One of the unique aspects of the mTORC1 is its dependency on nutrient availability for its kinase activity. Withdrawal of glucose or amino acids leads to rapid suppression of mTORC1 activity (28). The AMP-activated serine/threonine protein kinase (AMPK) is the major sensor of nutrient status found in all eukaryotes that is activated under low ATP conditions such as deprivation or hypoxia (28). One of the major downstream pathways regulated by AMPK is mTORC1 pathway. To examine if AMPK plays a role in the regulation of mTORC1 in lipotoxic cells, HepG2 cells were treated with palmitate and AMPK activator metformin (2 mM). AMPK phosphorylation (Thr172) and phospho-P70S6 (T389) kinase levels were studied in palmitate- and palmitate- + metformin-treated cells. Palmitate decreased the pAMPK-Thr172 levels and increased the p-P70S6K (T389) levels, hence suggesting a crosstalk between AMPK downregulation and mTORC1 activation in lipotoxic cells (Fig. 3, *A*–*C*). Activation of AMPK by metformin in lipotoxic cells increased the phosphorylation of AMPK-Thr172 and blocked the palmitate-induced activation of mTORC1–P70S6K axis (Fig. 3). These findings suggest that mTORC1–P70S6K axis is possibly activated through AMPK inhibition in lipotoxic conditions.

### Inhibition of mTORC1 decreased G9a levels and G9a-mediated repressive H3K9me2 mark at Atg7 and Beclin1 promoters

To gain further insight into the connection between mTORC1 activation and increased G9a-mediated H3K9me2 repressive mark at Atg7 and Beclin1 promoters in palmitate-treated cells, we tested whether the inhibition of mTORC1 has any impact on G9a-mediated repressive H3K9me2 mark at Atg7 and Beclin1 promoters.

**Figure 4:**
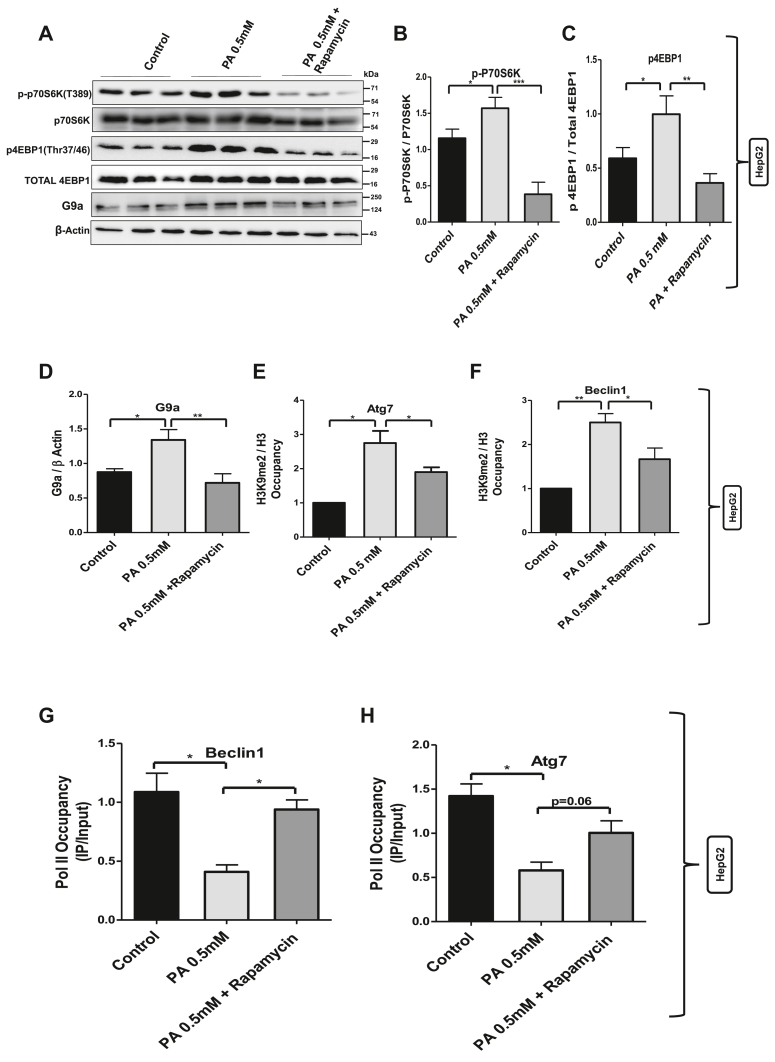
**Inhibition of mTORC1 reduced G9a-mediated repressive H3K9me2 mark at Atg7 and Beclin1 promoters.***A,* HepG2 cells treated with palmitate were pretreated (2 h) with mTORC1 inhibitor (100 nM) followed by Western blotting against Mtorc1 substrate p-P70S6K, p4E-BP1, and histone methyltransferase G9a in different treatment groups as indicated in the figure. *B*–*D,* bar graphs represent the densitometric ratio p-P70S6K with total P70S6K, p4E-BP1 with total 4E-BP1, and G9a with its corresponding β-actin (∗ indicates *p* < 0.05, ∗∗ indicates *p* < 0.01, and ∗∗∗ indicates *p* < 0.001). *E* and *F,* chromatin immunoprecipitation (ChIP) using H3K9me2 antibody and normalized to its corresponding total H3 followed by real-time PCR in different treatment groups as indicated in the figure for Atg7 and Beclin1 (∗ indicates *p* < 0.05 and ∗∗ indicates *p* < 0.01). *G* and *H,* chromatin immunoprecipitation analysis of polymerase II occupancy at Atg7 and Beclin1 in control and palmitate and palmitate- + rapamycin-treated cells. Error bars (wherever applicable in the figure) represent standard deviations from three independent experiments. mTORC1, mammalian target of rapamycin complex 1.

We used rapamycin (100 nM) to inhibit the mTORC1 hyperactivity in palmitate-treated cells. Rapamycin effectively downregulated the mTORC1 activity in palmitate-treated cells as evident by the decreased phosphorylation of its downstream targets: P70S6 kinase and 4E-BP1. This inhibition of mTORC1 activity was associated with decreased G9a protein levels in palmitate-treated cells (Fig. 4, *A*–*D*). ChIP experiments using H3K9me2 antibody show decreased occupancy of G9a-mediated repressive H3K9me2 mark at the promoter regions of Beclin1 and Atg7 in palmitate- + rapamycin-treated cells as compared with palmitate-treated cells (Fig. 4, *E* and *F*), thereby suggesting that increased G9a-mediated repressive H3K9me2 mark at the promoter regions of Beclin1 and Atg7 in palmitate-treated cells could be the result of mTORC1-mediated increased G9a expression and that targeting of this mTORC1–G9a axis could restore the expression of Atg7 and Beclin1 and hence autophagic machinery. Furthermore, RNA pol II occupancy, a readout of active transcription, increased at the promoter regions of Beclin1 and Atg7 in palmitate- + rapamycin-treated cells as compared with palmitate-treated cells only (Fig. 4, *G* and *H*), which again testify the importance of mTORC1–G9a axis in the regulation of autophagy in lipotoxic cells.

### Inhibition of mTORC1–G9a–H3K9me2 axis restored the expression of Atg7 and Beclin1 and increased autophagic flux

**Figure 5:**
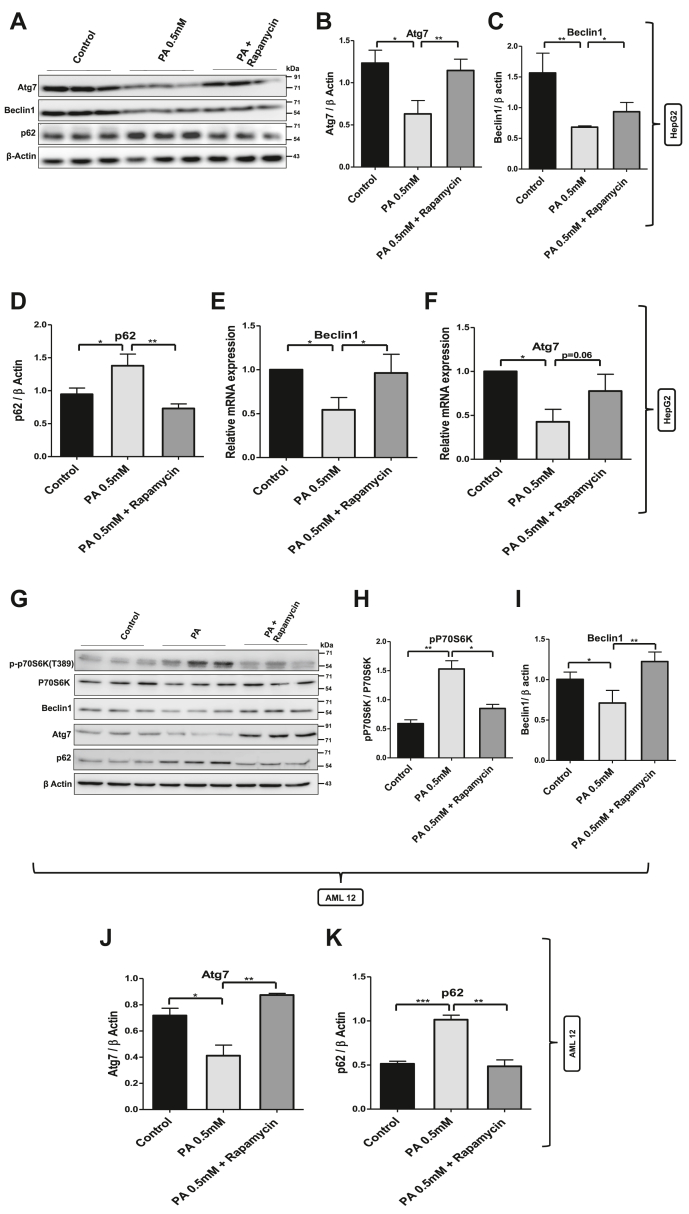
**Inhibition of mTORC1 restored the expression of Atg7 and Beclin1.***A,* HepG2 cells were pretreated for 2 h with mTORC1 inhibitor rapamycin (100 nM) and then treated with 0.5 mM palmitate for further 24 h. Cells were lysed and subjected to immunoblotting using antibodies against Atg7, Beclin1, and SQSTM1/p62 in different treatment groups as indicated in the figure (∗indicates *p* < 0.05 and ∗∗ indicates *p* < 0.01). *B*–*D,* represent the densitometric quantification of Atg7, Beclin1, and p-P70S6K with total P70S6K. *E* and *F,* mRNA level of autophagy genes Beclin1 and Atg7 in different treatment groups as indicated in the figure (∗ indicates *p* < 0.05). *G,* AML 12 cells pretreated with mTORC1 inhibitor rapamycin (100 nM) for 2 h were treated with 0.5 mM palmitate for 24 h. Cells were lysed and subjected to immunoblotting using antibodies as indicated in different treatment groups. *H*–*K,* the bar graphs represent the densitometric analysis along with statistical significance of corresponding immunoblots (∗ indicates *p* < 0.05, ∗∗ indicates *p* < 0.01, and ∗∗∗ indicates *p* < 0.001). Error bars (wherever applicable in the figure) represent standard deviations from three independent experiments. mTORC1, mammalian target of rapamycin complex 1.

Since inhibition of mTORC1 activity in palmitate-treated cells resulted in the decreased expression of histone methyltransferase G9a and G9a-mediated repressive H3K9me2 mark at Atg7 and Beclin1 promoters, we tested whether downregulation of G9a is associated with restoration of autophagy in palmitate-treated HepG2 cells. Western blot results showed that mTORC1 inhibition in palmitate-treated cells restored the protein levels of Beclin1 and Atg7, which was further associated with decreased levels of SQSTM1/p62, an autophagic cargo, thereby suggesting restoration of autophagy by mTORC1 inhibition in palmitate-treated cells (Fig. 5, *A*–*D*). We also checked the mRNA level of Atg7 and Beclin1 using quantitative RT–PCR (qRT–PCR) and observed that mTOR inhibition in palmitate-treated cells restored the mRNA levels of Beclin1 and Atg7 (Fig. 5, *E* and *F*). In order to understand that the aforeobserved phenomenon is not HepG2 specific, we tested whether inhibition of mTORC1 will lead to restoration of Atg7 and Beclin1 in immortalized mouse hepatocytes and murine lipotoxic cell model AML 12 cells. The results are consistent with HepG2 data. Our results with AML 12 cells indicate that mTORC1 activation upon palmitate treatment downregulated the expression of autophagy genes Atg7 and Beclin1 and increased the accumulation of autophagic cargo SQSTM1/p62, thereby suggesting the autophagic blockade in lipotoxic AML 12 cells. Inhibition of mTORC1 in lipotoxic mouse hepatocytes restored the levels of Atg7 and Beclin1, which was further associated with decreased accumulation of SQSTM1/p62 (Fig. 5, *G*–*K*).

### Inhibition of G9a alone restores the expression of genes involved in autophagosome formation and the process of autophagy

**Figure 7:**
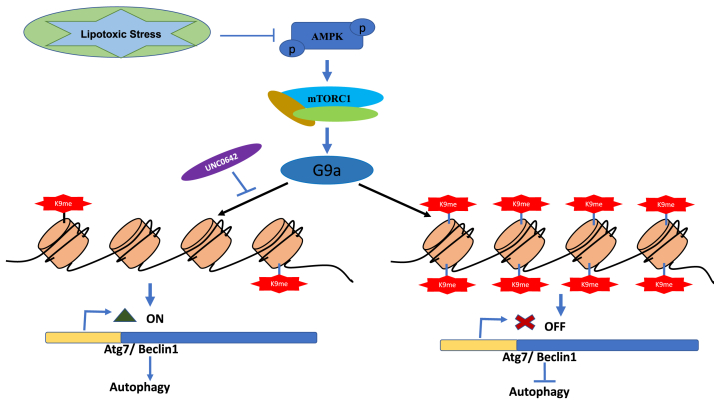
**Model showing the epigenetic connection of autophagy in lipotoxic human hepatoma cell.** mTORC1-mediated histone methyltransferase G9a negatively regulates autophagy by increasing repressive H3K9me2 mark at the promoter regions of autophagy genes in palmitate-treated cells and that the targeting of mTORC1–G9a–H3K9me2 axis restores autophagy in lipotoxic conditions in human hepatoma cells. mTORC1, mammalian target of rapamycin complex 1.

Since histone methyltransferase G9a is at the core of this mTORC1–G9a–H3K9me2 axis and our data point out to the important role played by this axis in the regulation of autophagy, we tested whether inhibition of G9a alone in palmitate-treated HepG2 cells could result in the restoration of ARGs. Interestingly, inhibition of G9a using UNC-0642 (10 μM) increased the LC3II/β-actin ratio and restored the expression levels of Atg7 and Beclin1 to that of control cells (Fig. 6, *A*–*E*). Furthermore, inhibition of G9a decreased the repressive H3K9me2 mark at Atg7 and Beclin1 promoter regions (Fig. 6, *F* and *G*). To test whether this mechanism is conserved, we used murine lipotoxic cellular model AML 12 cells. Our results show increased G9a-mediated H3K9me2 marks at promoter regions of Atg7 and Beclin1 in palmitate-treated cells. Inhibition of G9a using G9a-specific inhibitor UNC-0642 (10 μM) in lipotoxic AML 12 cells restored the expression of Atg7 and Beclin1 protein levels (Fig. 6, *H*–*J*) and reduced H3K9me2 levels at the promoter regions of Atg7 and Beclin1 (Fig. 6, *K* and *L*). Taken together, these results suggest that mTORC1–G9a–H3K9me2 axis negatively regulates autophagic process and that targeting this axis either by inhibiting mTORC1 or G9a restores the expression of genes involved in autophagosome formation and the process of autophagy (Fig. 7).

## Discussion

The adaptive and pathological changes occur at several levels within the cellular system when exposed chronically to free fatty acids. These changes serve to fine tune the complex cellular machinery controlling the cellular metabolism to a new fate. The evolution of these changes and their crosstalk is the determining factor in response to the changes. To understand the nature of these changes, we studied human hepatoma HepG2 cells and mouse hepatocyte AML 12 cells under lipotoxic conditions. Analysis of functional parameters, gene expression, and epigenetic alterations were combined to provide a comprehensive insight into the lipotoxic situation in hepatic cells. The analysis revealed mTORC1–G9a–H3K9me2 axis as key regulatory pathway of autophagy in lipotoxic conditions.

Lipotoxicity is the part of metabolic syndrome that results from the accumulation of lipids and their intermediates in nonadipose tissue, such as liver, kidney, heart, and skeletal muscle leading to cellular dysfunction and cell death (29). Lipotoxicity is the key pathogenic process in NAFLD and correlates with hepatic inflammation and fibrosis (30). Recent studies have shown autophagy as a new pathway for lipid metabolism and that the defective autophagy or inhibition of autophagy leads to the development of hepatosteatosis in mice (31, 32). Furthermore, autophagy has been shown to be impaired in liver from both NAFLD patients and murine models of NAFLD (33). However, the mechanism of defective autophagy in NAFLD is not well understood. Although autophagy is seen as cytoplasmic event, recent reports suggest great interplay of events between the cytoplasm and the nucleus for the regulation of autophagy program, wherein studies revealed that transcriptional and epigenetic regulation within the nucleus are critical for the autophagic process; however, the mechanistic aspects of transcriptional and epigenetic regulation of autophagy in lipotoxicity are not fully understood (23). Euchromatic histone–lysine N methyltransferase (G9a) is the histone methyltransferase that catalyze the synthesis of monomethylated and dimethylated lysine 9 on histone 3 (H3K9me1 and H3K9me2). H3K9 methylation mediates heterochromatin formation and is involved in gene silencing (34). G9a is ubiquitously expressed in somatic cells and plays role in various biological processes, such as stem cell differentiation, lymphocyte differentiation, and tumor cell growth, and is highly expressed in a variety of human cancers, such as leukemia, prostate, lung, and hepatocellular carcinoma (35). Furthermore, recent studies identified G9a as an epigenetic regulator of autophagy that participates in the repression of key genes involved in autophagosome formation under normal growth conditions (36). However, the role of G9a in the regulation of autophagy in lipotoxic conditions such as NAFLD and obesity is yet to be elucidated. Therefore, we hypothesized that histone methyltransferase G9a might be repressing the key genes involved in autophagy and hence regulates autophagy in lipotoxic cells. To test our hypothesis, we used lipotoxic human hepatoma HepG2 cells and mouse hepatocyte AML 12 as cellular models and palmitate complexed with albumin as lipotoxic agent. Our results showed that exposure of palmitate induced significant lipotoxicity and lipid accumulation (Fig. 1, *A*–*C*). The increased lipogenesis and lipid accumulation was associated with downregulation of autophagy as suggested by decreased LC3II/actin ratio in lipotoxic cells as compared with untreated control cells (Fig. 1, *D* and *G*). The decrease in LC3II levels suggests a defect in autophagosome formation in lipotoxic cells. This was also accompanied by increased accumulation of autophagic cargo p62/SQSTM1 (Fig. 1, *D* and *H*), which reflects the decrease in autophagic flux or execution. p62/SQSTM1 is a multifunctional adapter protein involved in various cell signaling pathways with significant implications in the development of oxidative stress–mediated cellular damage and tumorigenesis (37). Our observation of defective autophagy in lipotoxic cells was further supported by the decreased mRNA as well as protein levels of key autophagy gene Atg7 (Fig. 1, *D*, *E* and *I*), whose deficiency leads to impaired autophagy, accumulation of p62/SQSTM1, and development of multiple liver tumors (38). Beclin1, a mammalian ortholog of Atg6/vacuolar protein sorting 30 in yeast and a Bcl-2-homology-s domain only protein, is another key autophagic gene playing critical role in autophagy and mediates the crosstalk between apoptosis and autophagy (39). The nucleation step of autophagy requires the essential service of Beclin1. The embryonic phenotype of Beclin1 null mice (beclin1^−/−^) dies in early embryonic development, which is even more severe than other autophagy-gene–deficient mice (40). We tested the expression of Beclin1 in palmitate-treated cells and found that palmitate significantly decreased both mRNA and protein levels of Beclin1 (Fig. 1, *D*, *F* and *I*), thus providing further evidence that autophagy is impaired in palmitate-treated cells.

One of the processes that has been consistently linked with suppressed autophagy is mTORC1 activation (41, 42). mTORC1 positively regulates cell growth and proliferation by inducing many anabolic processes, such as biosynthesis of lipids, proteins, and cellular organelles, and negatively regulates catabolic processes such as autophagy (12, 42). We measured the phosphorylation of one of the substrates of mTORC1: P70S6 kinase (T389) and found that autophagy blockade in lipotoxic cells is associated with mTORC1 activation (Fig. 2). mTORC1 plays key roles not only in growth control and cellular proliferation but also in metabolism. Recent studies across eukaryotes have revealed that mTORC1 is acutely sensitive to rapamycin and significantly regulated by nutrients and AMPK (28). AMPK is linked to mTORC1 control by two separate pathways. In one pathway, AMPK phosphorylates tuberous sclerosis 2, which would indirectly cause inhibition of mTORC1 by deactivation of Rheb GTPase (43). Other pathways include the phosphorylation of mTORC1 binding partner raptor on two well-conserved serine residues, and this phosphorylation induces 14-3-3 protein binding to raptor and results in the inhibition of mTORC1 (44). We also wanted to check the status of AMPK activity in lipotoxic cells and understand its correlation with mTORC1 activity. Therefore, we measured the pAMPK in lipotoxic cells. We found decreased pAMPK in palmitate-treated cells as compared with untreated HepG2 control cells (Fig. 3). Next, we used the metformin, an activator of AMPK, in palmitate-treated cells. The use of metformin in lipotoxic cells resulted in the activation of AMPK and downregulation of mTORC1 (Fig. 3), hence suggesting that both APMK and mTORC1 are dysregulated during lipotoxic conditions in liver cells. AMPK inhibition ensures mTORC1 activation and autophagy blockade in lipotoxic cells. Our data point to a greater role played by AMPK–mTORC1 axis in the regulation of autophagy in lipotoxic cells, which requires further mechanistic studies.

Another phenomenon that has been shown to put brakes on autophagy is G9a-mediated histone methylation (36); however, the underlying mechanism seems quite complicated and also cell type dependent. Fewer reports have linked mTOR and histone methyltransferase G9a function. One such study suggests that histone methyltransferase G9a negatively regulates AMPK/mTOR pathway in bladder cell carcinoma (45). Other reports suggest that G9a promotes cell proliferation in gastric cancer and suppresses autophagy by activating mTOR (46). However, there are other contrasting reports suggesting that mTORC1 induces histone methyltransferase G9a *via* unknown mechanism (36). Therefore, it is important to understand the crosstalk between mTORC1 and G9a in lipotoxic states. In addition to monomethylation and dimethylation of H3K9, G9a can directly recruit DNA methyltransferases to gene promoters resulting in the methylation of CpG islands and gene repression (47). Therefore, we measured histone methyltransferase G9a in cells and G9a-mediated repressive histone methylation mark (H3K9me2) at Atg7 and Beclin1 promoters. Quite interestingly, mTORC1 activation and autophagy blockade were associated with increased protein level of G9a (Fig. 2) and increased occupancy of G9a-mediated H3K9me2 at Atg7 and Beclin1 promoters in lipotoxic cells (Fig. 2). These results provide experimental evidence that histone methyltransferase G9a-mediated gene repression might be playing a key role in the regulation of autophagy. Furthermore, RNA polymerase II occupancy at the promoter regions of Beclin1 and Atg7 was decreased in palmitate-treated cells, which again supports the evidence that transcription of these genes is reduced in lipotoxic cells. In order to further delineate the events specifically, we inhibited the mTORC1 and G9a in lipotoxic cells in separate experimental setups. Inhibition of mTORC1 in lipotoxic cells decreased the G9a levels (Fig. 4) and G9a-mediated H3K9me2 occupancy at Atg7 and Beclin1 promoters (Fig. 4, *E* and *F*). Furthermore, inhibition of the mTORC1 in palmitate-treated cells increased the RNA polymerase II levels at the promoter regions of Atg7 and Beclin1 (Fig. 4, *G* and *H*). We also checked the relative gene expression of Atg7 and Beclin1 in different experimental groups, and our results indicate increased mRNA and protein levels of Atg7 and Beclin1 in palmitate + rapamycin-treated cells as compared with palmitate-treated cells (Fig. 5). These results clearly show that G9a-mediated gene repression represents a key step that negatively regulates the process of autophagy in lipotoxic cells and suggested that G9a is the key regulator of autophagy.

Since our results point toward a key role for G9a in regulation of autophagy, we decided to test if inhibition of G9a alone could restore the expression of ARGs. Interestingly, we found that pharmacological inhibition of G9a increased the LC3II expression, and our findings are consistent with the previous findings where it was reported that inhibition of G9a was sufficient to induce LC3II formation, an indicative of autophagosome biogenesis (36). More interestingly, G9a inhibition in lipotoxic cells decreased the G9a-mediated H3K9me2 mark at the promoter regions of Atg7 and Beclin1 and restored the expression levels of Atg7 and Beclin1, the key genes involved in autophagy process (Fig. 6). Previous studies suggest that the autophagic flux downstream of G9a inhibition still required mTORC1 inhibition, suggesting that mTORC1 pathway is required for the completion of autophagy process (36). However, our study identified histone methyltransferase G9a as a key epigenetic regulator of autophagy that participates in the repression of key genes involved in autophagosome formation in lipotoxic cells (Fig. 7). Furthermore, G9a-mediated regulation of autophagy is also evolutionary conserved, as several of the autophagy genes were found to be induced following G9a inhibition in *Drosophila melanogaster* (48). Taken together, our results indicate that mTORC1–G9a–H3K9me2 axis negatively regulates autophagy in lipotoxic hepatic cells.

The inhibition of mTORC1 is considered as the potential treatment for lipotoxicity in nonalcoholic steatohepatitis; however, conflicting interpretations have been proposed. In addition, direct targeting of mTORC1 is known to have various off target effects. Therefore, a good understanding of the mTOR pathway’s connectivity, its downstream effectors are of great importance toward our ability for genetic and pharmacological interventions for the amelioration of metabolic diseases, such as diabetes, obesity, and NAFLD. Our results identified histone methyltransferase G9a as a potential downstream effector of mTORC1 that is involved in the repression of key genes of the autophagy process under lipotoxic conditions such as NAFLD (Fig. 7). Hence, our study, on one hand, signifies the therapeutic potential of G9a that can be tested in preclinical models of NAFLD and on the other hand, highlight the need to understand the loci-specific recruitment of G9a and sequence-specific regulation of G9a by mTORC1 for better understanding of the disease outcome in lipotoxicity.

## Experimental procedures

Human hepatoma HepG2 cell line was obtained from National Centre for Cell Science, Department of Biotechnology, India. Immortalized mouse hepatocyte AML 12 cells were obtained from American Type Culture Collection. HepG2 Cells were grown and maintained in minimum essential medium containing l-glutamine, glucose (3.5 g/l), 15 mM Hepes, 200 U/ml penicillin, 270 μg/ml streptomycin, 1% (v/v) nonessential amino acids, 10% (v/v) obtained from Sigma–Aldrich, and 10% (v/v) fetal bovine serum, obtained from Gibco at 37 °C in a humidified atmosphere of 5% CO_2_. AML 12 cells were grown in Dulbecco's modified Eagle's medium F-12 containing 200U/ml penicillin, 270 μg/ml streptomycin, 1% (v/v), 10% (v/v) fetal bovine serum, obtained from Gibco and supplemented with Insulin Transferrin Selenium (ITS) obtained from Sigma Aldrich, St Louis, MO. For all experiments cells were used at density of 7 × 10^5^ cells/6 well plate or 1.2 × 10^6^ cells/60 mm dish, unless otherwise mentioned. All the studies were conducted using 70 to 80% confluent cells, which were treated with indicated concentrations of palmitate (Sigma Aldrich, St Louis, MO) for various time points as indicated. For mTORC1 inhibition with rapamycin (100 nM), AMPK activation with Metformin (2 mM) (Sigma Aldrich, St Louis, MO), cells were pre-treated 2 h prior to palmitate exposure. For G9a inhibition experiments, HepG2 and AML 12 cells were pretreated with indicated concentration of G9a inhibitor UNC 0642 (Sigma Aldrich, St Louis, MO) for 2 h and 12 h respectively prior to palmitate exposure.

### FFA-BSA complex preparation

Palmitate was complexed to fatty acid free BSA (Sigma Aldrich, St Louis, MO) as mentioned previously (25). Briefly, 20% stock solution of fatty acid free BSA was prepared by dissolving 750 mg of BSA in 3.75 ml PBS (pH 7.4). To begin with, BSA was layered on top of the 2 to 2.5 ml PBS. BSA was allowed to dissolve on its own (without stirring) at 4 °C. The final volume was adjusted to 3.75 ml. For 20 mM Palmitic acid solution, 5.6 mg of sodium Palmitic acid was added to 1 ml water pre-heated to 70 °C. The mixture was incubated at 70 °C for 20 to 30 min with constant vortexing. FFA-BSA complex was prepared by mixing 1 ml of 20 mM Palmitic acid solution to 3.3 ml of 20% BSA (pre-warmed at 37 °C). Complex formed was immediately added to 15.7 ml Dulbecco's modified Eagle's medium prewarmed to 37 °C. Final solution prepared had 1 mM palmitic acid. Solution was sterile filtered and stored at 4 °C.

### 3-(4,5-Dimethylthiazol-2-yl)-2,5-diphenyltetrazolium bromide assay

Cytotoxicity test was done to identify the cytotoxic potential of palmitate as mentioned previously (25). Briefly, cells were suspended in 96-well plates at a density of 0.7 × 10^4^ cells per well. The cells were incubated for 24 h with different concentrations of palmitate (0.1–0.5 mM). After 24 h, medium was removed, and the cells were incubated in fresh medium containing 250 μg/ml 3-(4,5-dimethylthiazol-2-yl)-2,5-diphenyltetrazolium bromide for 3 h at 37 °C. Cytotoxicity/cell viability was evaluated by assaying the ability of mitochondria to catalyze the reduction of thiazolyl blue tetrazolium bromide (3-(4,5-dimethylthiazol-2-yl)-2,5-diphenyltetrazolium bromide) to a formazan salt by mitochondrial dehydrogenases.

### RNA extraction, complementary DNA synthesis, and qRT–PCR

The mRNA level of different target genes was studied using RT–qPCR. Total RNA from control and treated cells was isolated using TRIzol reagent. For complementary DNA synthesis, 2 μg of total RNA was processed using RevertAid first strand complementary DNA synthesis kit (Thermo Fisher Scientific) according to the manufacturer’s instructions. qRT–PCR was performed in 7500 Real Time PCR System (Applied Biosystem) using SYBR Green PCR master mix (Thermo Fisher Scientific). The PCR amplification was carried out using the primers listed in Table S1. Relative RNA levels of corresponding genes were normalized to GAPDH mRNA (an endogenous control), and relative expression levels were calculated using comparative ΔΔCt method.

### Protein isolation and Western blotting

Human hepatoma HepG2 cells and AML 12 cells treated with palmitate, with or without rapamycin and UNC-0642 for indicated time point(s), were scrapped and suspended in radioimmunoprecipitation assay buffer containing protease/phosphatase inhibitor cocktail and phenylmethylsulfonyl fluoride. Cells were sonicated for 30 s using ultrasonic processor (Vibracell) with 40% amplitude and 5 s ON and 10 s OFF cycle. The lysate was centrifuged at 14,000 rpm for 30 min at 4 °C to remove cellular debris. Protein concentrations were determined by Bradford assay. For Western blotting, 30 μg of protein were denatured at 100 °C for 5 min in Laemmli buffer. Protein samples were resolved on 7 to 18% SDS gels at 100 V. Proteins were electrotransferred to polyvinylidene difluoride membrane (GE Healthcare Life Science) using Bio-Rad Mini Transblot Electrophoretic transfer Cell. Membranes were blocked in 5% fat-free milk in 50 mM Tris, pH 8.0 with 150 mM sodium chloride and 0.1% Tween-20 for 2 h. Rabbit: anti-LC3II, anti-Beclin1, anti-Atg7, anti-pP70S6 kinase, anti-P70S6 kinase, anti-G9a, anti-pAMPK-Thr172, anti-p4-EBP1, anti-4-EBP1 (Cell Signalling Technology), anti-SQSTM1/p62 (Santa Cruz Biotechnologies), mouse: anti-β-actin (Sigma–Aldrich), antibodies were used as primary antibodies in 3% BSA overnight at 4 °C. Anti-H3K9me2, anti-H3, and anti-Pol II antibodies obtained from abcam were used for ChIP experiments. Goat anti-rabbit and goat antimouse immunoglobulin G antibodies conjugated with horseradish peroxidase (Cell Signalling Technology) were used as secondary antibodies. Chemiluminescence was detected by ECL Start Western blotting detection reagent (GE Healthcare Life Science) and visualized by Odyssey CLx Imaging System. Densitometric measurement of the bands was performed using ImageJ software (National Institutes of Health and the Laboratory for Optical and Computational Instrumentation, University of Wisconsin), and the band detection was within the linear range.

### ChIP assay

ChIP assays were performed in cells treated with palmitate, with or without rapamycin and UNC-0642 for indicated time point(s). Cells were crosslinked using 1% formaldehyde for 15 min followed by quenching using 125 mM glycine for 5 min. Cells were lysed, and cross-linked chromatin was sonicated for 5 min using ultrasonic processor (Vibracell) with 40% amplitude and 15 s ON and 30 s OFF cycle to obtain DNA fragments of 400 to 700 bp in size. Immunoprecipitations were carried out using anti-H3K9me2 antibody, anti-H3 antibody, and anti-Pol II antibody. All the ChIP experiments were performed thrice with similar results. qPCR was performed using SYBR Green reagent (Thermo Scientific) according to the manufacturer’s protocol using Real Time Cycler (Applied Biosystems). The results shown are based on three independent experiments with standard errors. Primers used in the ChIP experiment are listed in Tables S2 and S3.

### Statistical analysis

Data are presented as mean ± SD from three independent experiments. Statistical comparisons between the groups were determined by using one-way ANOVA followed by Bonferroni multiple comparison test using GraphPad Prism 8.0.1 statistical software. *p* < 0.05 was considered as statistically significant.

## Data availability

All the data described in this study are contained within the article.

## Supporting information

This article contains supporting information.

## Conflict of interest

The authors declare that they have no conflict of interest with the contents of this article.
