# Supplementary material for: The mTORC1–G9a–H3K9me2 axis negatively regulates autophagy in fatty acid–induced hepatocellular lipotoxicity
Source: J Biol Chem. 2023 Jan 21;299(3):102937. doi: 10.1016/j.jbc.2023.102937 (PMC9957777; doi:10.1016/j.jbc.2023.102937)
Supplement: Supplemental Table 1 [file mmc1.pdf]

Supplemental Table 1

List of Primers for qRT-PCR in HepG2 cells

Gene	Forward Primer	Reverse primer
**Atg7**	5'-ACCCAGAAGAAGCTGAACGA-3'	5'-CTCATTTGCTGCTTGTTCCA-3'
**Atg5**	5'-TGGGATTGCAAAATGACAGA-3'	5'-TTCCCCATCTTCAGGATCAA-3'
**Beclin1**	5'-AGGTTGAGAAAGGCGAGACA-3'	5'-AATTGTGAGGACACCCAAGC-3'
**SREBP1c**	5'-TCAGCGAGGCGGCTTTGGAGCAG-3'	5'-CATGTCTTCGATGTCGGTCAG
**FAS**	5'-CGGTACGCGACGGCTGCCTG-3'	5'-GCTGCTCCACGAACTCAAACACCG-3'
**SCD1**	5'-GGCTCCCAAGTGTAGCAGAG-3'	5'-TACCACCACCACCACCATTAC-3'
**GAPDH**	5'-TGACTCCGACCTTCACCTTC-3'	5'-CTCTCTGCTCCTCCTGTTCG-3'
