# Supplementary material for: The mTORC1–G9a–H3K9me2 axis negatively regulates autophagy in fatty acid–induced hepatocellular lipotoxicity
Source: J Biol Chem. 2023 Jan 21;299(3):102937. doi: 10.1016/j.jbc.2023.102937 (PMC9957777; doi:10.1016/j.jbc.2023.102937)
Supplement: Supplemental Table 2 [file mmc2.pdf]

Supplemental Table 2

List of Primers for ChIP-qPCR in HepG2 cells

Gene	Forward Primer	Reverse primer
**Atg7**	5'-CTGCCAGTTTCTGGGTGGTC-3'	5'-GTGCCAGAGACAGCAAATCCA-3'
**Atg5**	5'-CACTTCCGCCCTCTGGTATC-3'	5'-GAGAGGTTTGGTCGCGAGTT-3'
**Beclin1**	5'-GTCACCCAAGTCCGGTCTAC-3'	5'-CTTGTTCATCCGCTGAAGCC-3'
