# Supplementary material for: The mTORC1–G9a–H3K9me2 axis negatively regulates autophagy in fatty acid–induced hepatocellular lipotoxicity
Source: J Biol Chem. 2023 Jan 21;299(3):102937. doi: 10.1016/j.jbc.2023.102937 (PMC9957777; doi:10.1016/j.jbc.2023.102937)
Supplement: Supplemental Table 3 [file mmc3.pdf]

Supplemental Table 3

List of Primers for ChIP-qPCR in AML 12 cells

Gene	Forward Primer	Reverse Primer
Atg7	5' GTGTAAATATGAGGAGGACA-3'	5' GGCTACTGTTCTTACCAGCC-3'
Beclin1	5' CTCTGTGAAGTGGATGCCAG-3'	5' AGACCTCCAGAGTTCCCATC-3'
